# 

**Published:** 1883-11

**Authors:** 


					﻿j ESTABLISHED IN 1S5B.
££ SSSo. 8>/ NOVEMBER, 1883.	vS'KS^
---------------------------------
ATLANTA
MEDICAL REGISTER.
(ATLANTA MEDICAL AND SURGICAL JOURNAL.)
ZDITED STT
J. H. LOGAN, A.M., M.D.
H.H. DICKSON, PUBLISHER. TERMS, $1.50 A YEAR IN ADVANCE.
ATLANTA, GEORGIA':
i.E. E. DICKSON, BOOK AND JOB PRINTER AND BOOKBINDER.
.1883.
				

